# Metabotropic glutamate receptor 1 (mGluR1) and 5 (mGluR5) regulate late phases of LTP and LTD in the hippocampal CA1 region *in vitro*

**DOI:** 10.1111/j.1460-9568.2008.06109.x

**Published:** 2008-03-01

**Authors:** Sergey Neyman, Denise Manahan-Vaughan

**Affiliations:** 1 Institute for Physiology of the Charité, Synaptic Plasticity Research Group, Humboldt University Berlin, Germany; 2 Department of Experimental Neurophysiology, Medical Faculty, Ruhr University Bochum MABF 01/551, Universitaetsstr. 150, 44780 Bochum, Germany; 3 International Graduate School of Neuroscience, Ruhr University Bochum Germany

**Keywords:** anisomycin, CHPG, hippocampus, LY367385, MPEP, synaptic plasticity, Wistar

## Abstract

The group I metabotropic glutamate receptors, mGluR1 and mGluR5, exhibit differences in their regulation of synaptic plasticity, suggesting that these receptors may subserve separate functional roles in information storage. In addition, although effects *in vivo* are consistently described, conflicting reports of the involvement of mGluRs in hippocampal synaptic plasticity *in vitro* exist. We therefore addressed the involvement of mGluR1 and mGluR5 in long-term potentiation (LTP) and long-term depression (LTD) in the hippocampal CA1 region of adult male rats *in vitro*. The mGluR1 antagonist (S)-(+)-α-amino-4-carboxy-2-methylbenzene-acetic acid (LY367385) impaired both induction and late phases of both LTP and LTD, when applied before high-frequency tetanization (HFT; 100 Hz) or low-frequency stimulation (LFS; 1 Hz), respectively. Application after either HFT or LFS had no effect. The mGluR5 antagonist 2-methyl-6-(phenylethynyl)pyridine (MPEP), when given before HFT, inhibited both the induction and late phases of LTP. When given after HFT, late LTP was inhibited. MPEP, given prior to LFS, impaired LTD induction, although stable LTD was still expressed. Application after LFS significantly impaired late phases of LTD. Activation of protein synthesis may comprise a key mechanism underlying the group I mGluR contribution to synaptic plasticity. The mGluR5 agonist (R,S)-2-chloro-5-hydroxyphenylglycine (CHPG) converted short-term depression into LTD. Effects were prevented by application of the protein synthesis inhibitor anisomycin, suggesting that protein synthesis is triggered by group I mGluR activation to enable persistency of synaptic plasticity. Taken together, these data support the notion that both mGluR1 and mGluR5 are critically involved in bidirectional synaptic plasticity in the CA1 region and may enable functional differences in information encoding through LTP and LTD.

## Introduction

Evidence obtained using general and specific antagonists of group I metabotropic glutamate receptors (mGluRs) suggest that these receptors are critically important for both hippocampal synaptic plasticity *in vivo* and hippocampus-based learning (Manahan-Vaughan, 1997, Balschun *et al*., 1999; Manahan-Vaughan *et al*., 1998, 1999; Wu *et al*., 2001; Naie & Manahan-Vaughan, 2004, 2005; Manahan-Vaughan & Braunewell, 2005). Group I metabotropic glutamate receptors include mGluR1 and mGluR5, both of which are coupled to phospholipase C via Gq proteins and mediate phosphoinositide hydrolysis. Previous studies have shown that activation of mGluR1 results in an increase in intracellular calcium concentration, depolarization of CA1 pyramidal neurons and an increased frequency of spontaneous inhibitory postsynaptic potentials (Mannaioni *et al*., 2001). In contrast, activation of mGluR5 results in suppression of the calcium-activated potassium current (*I*_AHP_) and a potentiation of *N*-methyl-d-aspartate (NMDA) receptor currents (Jia *et al*., 1998; Attucci *et al*., 2001; Mannaioni *et al*., 2001). The different functional contributions of group I mGluR subtypes to cellular excitability may have distinct consequences for synaptic plasticity and memory processes.

In previous work we have demonstrated that selective antagonism of either mGluR1 or mGluR5 results in a significant impairment of both induction and maintenance of long-term potentiation (LTP) in freely moving adult rats, an impairment that is associated with disruption of spatial memory (Naie & Manahan-Vaughan, 2004, 2005; Manahan-Vaughan & Braunewell, 2005). The impairments of synaptic plasticity mediated by mGluR1 *in vivo* may be mediated by alterations in intracellular calcium release or disruption of other mGluR1-mediated processes such as depolarization of CA1 pyramidal neurons, depression of the slow afterhyperpolarization (Ireland & Abraham, 2002; Ireland *et al*., 2004) and an increase in the frequency of spontaneous inhibitory postsynaptic potentials (Mannaioni *et al*., 2001; Rae & Irving, 2004). Transgenic mice lacking mGluR5 show abnormal hippocampal LTP expression and impairments in both spatial learning and fear conditioning (Lu *et al*., 1997; Jia *et al*., 1998). Whereas impairment of LTP induction by antagonism of mGluR5 may be mediated by a reduction in NMDA receptor currents (Mannaioni *et al*., 2001), the impairment of LTP maintenance may be associated with disruption of expression mechanisms of LTP, such as protein synthesis (Frey *et al*., 1988).

Reports that group I mGluR antagonism or deletion consistently impairs hippocampal plasticity *in vivo* are strikingly consistent (Aiba *et al*., 1994; Manahan-Vaughan, 1997; Balschun *et al*., 1999; Balschun & Wetzel, 2002; Naie & Manahan-Vaughan, 2004, 2005). Other reports support a role for group I mGluRs in synaptic plasticity *in vitro* (Lu *et al*., 1997; Raymond *et al*., 2000; Miura *et al*., 2002; Hou & Klann, 2004; Volk *et al*., 2006). However, it has also been reported that mGluR antagonism does not affect synaptic plasticity in the hippocampal slice preparation (Chinestra *et al*., 1993; Manzoni *et al*., 1994; Selig *et al*., 1995; Thomas & O'Dell, 1995; Fitzjohn *et al*., 1999; Doherty *et al*., 2000), leading to the postulate that plasticity induced specifically with high-frequency stimulation (e.g. 100 Hz, theta burst) and low-frequency stimulation (LFS; single pulses, 1–2 Hz) is NMDAR- but not mGluR-dependent. We suspected that these differences may derive on the one hand from the specificity of mGluR ligands used and on the other hand from the duration of observations in *in vitro* studies compared to *in vivo* studies. *In vitro* analysis of synaptic plasticity is usually conducted for 60–90 min after induction of synaptic plasticity; however, the effects of group I antagonism on synaptic plasticity *in vivo* typically appear 2–3 h after induction of plasticity (Manahan-Vaughan, 1997). This suggests that the failure to find an effect of group I antagonism on synaptic plasticity *in vitro* may be an issue of detectability. In line with this, one of the rare *in vitro* studies that addressed effects of group I antagonism on late phases of LTP found that mGluR5 antagonism is effective (Francesconi *et al*., 2004).

This study aimed to examine whether antagonism of mGluR1 or mGluR5 affects late phases of either LTP or long-term depression (LTD) in hippocampal slice preparation. As group I mGluRs can influence NMDA receptor currents (Mannaioni *et al*., 2001) we additionally investigated whether differences arise if antagonism occurs during or after the plasticity induction phase. By this means we aimed to clarify to what extent an interaction with the NMDA receptor may contribute to the involvement of group I mGluRs in persistent synaptic plasticity. We also investigated whether the effects of group I mGluRs on late phases of plasticity may be mediated by protein synthesis.

## Materials and methods

### In vitro *electrophysiology*

Seven- to eight-week-old male Wistar rats were anaesthetized with ether and then decapitated. Brains were dissected in ice-cold artificial cerebrospinal fluid.

Immediately after preparation, slices (400 µm) were placed on a nylon net in a 2-mL circulation chamber at the interface between the incubation medium and a humidified atmosphere of 95% O_2_ and 5% CO_2_; the chamber was continuously perfused (at a constant flow rate of 3 mL/min) with an oxygenated Ringer's solution (in mm: NaCl, 124; KCl, 4.9; KH_2_PO_4_, 1.2; MgSO_4_, 1.3; CaCl_2_, 2.5; NaHCO_3_, 25.6; and d-glucose, 10) at 35 °C. Following 30 min equilibration, the slices were submerged by filling the chamber to a volume of 3 mL with warmed (35 °C) O_2_/CO_2_ Ringer's solution. The flow rate was then adjusted to 0.8 mL/min.

Monopolar platinum-tipped silver chloride electrodes were positioned in the stratum radiatum of the CA1 region for stimulation and in the CA1 dendritic area for recording (Dunwiddie *et al*., 1978; Frey *et al*., 1988). The recording electrode was placed at a distance of ∼ 100 µm from the cell body layer. Typically recordings were taken from two hippocampal slices simultaneously one slice was used for experimental analysis while test pulses were applied to the second slice to monitor basal synaptic transmission and evaluate slice viability during the course of the experiment.

### Measurement of evoked potentials

Responses were evoked by stimulating at low frequency (0.025 Hz, 0.2 ms stimulus duration; 16 000 Hz sample rate). For each time point, five evoked responses were averaged. The slope of the field excitatory postsynaptic potential (fEPSP) was measured as the maximum slope through the five steepest points obtained on the first positive deflection of the potential. By means of input–output curve determination the maximum fEPSP slope was found for each individual animal, and all potentials employed as baseline criteria were evoked at a stimulus intensity which produced 40% of this maximum.

LTP was induced from one stimulation input only; the other input was used to generate test-pulse responses. LTP was induced with high-frequency tetanization (HFT, 100 Hz) comprising three stimulus trains, at 5-min intervals, of 100 pulses. Short-term potentiation (STP) was induced with HFT (100 Hz) comprising one stimulus train of 100 pulses. Persistent LTD was induced with LFS at 2 Hz (1200 pulses) whereas short-term depression (STD) was induced with LFS at 1 Hz (900 pulses).

### Compounds and drug treatment

The metabotropic glutamate receptor antagonists (R,S)-2-chloro-5-hydroxyphenylglycine (CHPG), LY367385 and 2-methyl-6-(phenylethynyl)pyridine (MPEP) were obtained from Tocris Cookson, Bristol, UK. The protein synthesis inhibitor anisomycin was obtained from Sigma-Aldrich, Germany.

### Data analysis

The baseline fEPSP data were obtained by averaging the response to stimulating the Schaffer collaterals, to obtain five sweeps at 0.025 Hz, every 5 or 15 min as described above. The data were then expressed as mean percentage preinjection baseline reading ± SEM. Statistical significance was estimated using anova with repeated measures, followed by *post hoc* Student's *t*-tests. Within-group and between-group analysis was conducted. The probability level interpreted as statistically significant was *P* < 0.05.

## Results

### Application of an mGluR1 antagonist prior to, but not after, HFT prevented LTP in the CA1 region

LY367385 is a highly selective antagonist of mGluR1 receptors. This compound antagonizes mGluR1α receptors in recombinant cells in the low micromolar range (IC_50_, 8.8 µm). It fails to interact with other mGluR subtypes up to 100 µm (Clark *et al*., 1997).

**Fig. 1:**
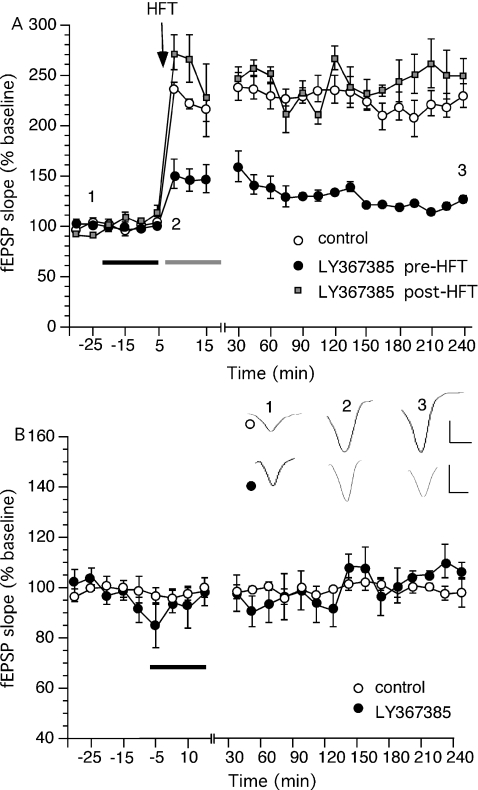
Application of an mGluR1 antagonist prior to, but not after, HFT prevented LTP in the CA1 region. (A) HFT (100 Hz) induced persistent LTP (which lasted for at least 4 h) in the CA1 region *in vitro*. Application of the mGluR1 antagonist LY367285 (100 µm), for 20 min prior to HFT, significantly prevented the induction and expression of LTD. Application of LY367285 (100 µm) for 20 min immediately after HFT had no effect. Bar indicates drug application before (black) or after (grey) HFT. (B) Application of LY367285 (100 µm) did not affect basal synaptic transmission compared to controls. Bar indicates drug application. Insets: evoked potentials obtained in the presence of vehicle or LY387385 (applied pre-HFT), at the timepoints noted: vertical bars, 2 mV; horizontal bars, 2 ms.

Application of LY367385 (100 µm) for 20 min immediately prior to HFT resulted in a significant inhibition of LTP in hippocampal slices (*n* = 5) compared to controls (*n* = 8; Fig. 1A; anova: within factor *F*_1,23_ = 36.466, *P* = 0.0001; between factor *F*_1,23_ = 8.214, *P* = 0.0001). A significant effect on the induction phase was evident (*P* < 0.05). In addition, the expression phase of LTP (late LTP, > 2 h) was markedly impaired compared to controls (Fig. 1A). LTP in control animals persisted for at least 4 h.

When LY367385 was applied after the tetanus (*n* = 8), no significant effect on the profile of LTP was seen (anova: within factor *F*_1,23_ = 1.823, *P* = 0.087; between factor *F*_1,23_ = 1.349, *P* = 0.164). LY367385 (100 µm) had no effect on basal synaptic transmission compared to controls (Fig. 1B). (anova: within factor *F*_1,23_ = 1.313, *P* = 0.1663, between factor *F*_1,23_ = 1.222, *P* = 0.2331). These data suggest that mGluR1 contributes to LTP processes by modulating the induction phase.

### Application of an mGluR5 antagonist either prior to or after HFT prevented LTP in the CA1 region

MPEP is a highly selective antagonist at mGluR5. This compound exhibits an IC_50_ of 36 nm at mGluR5 with no activity at any other mGluR subtype (Gasparini *et al*., 1999).

**Fig. 2:**
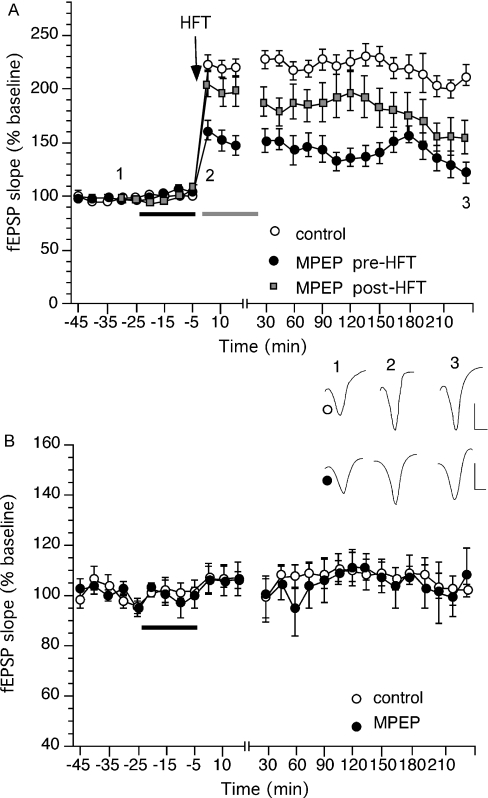
Application of an mGluR5 antagonist either prior to or after HFT prevented LTP in the CA1 region. (A) HFT (100 Hz) induced persistent LTP (which lasted for at least 4 h) in the CA1 region *in vitro*. Application of the mGluR5 antagonist MPEP (40 µm), for 20 min prior to HFT, significantly prevented both the induction and the expression of LTP. Application of MPEP (40 µm) for 20 min after HFT significantly prevented the expression of LTP beyond 2 h post-HFT. Bar indicates drug application before (black) or after (grey) HFT. (B) Application of MPEP (40 µm) did not affect basal synaptic transmission compared to controls. Bar indicates drug application. Insets: evoked potentials obtained in the presence of vehicle or MPEP (applied pre-HFT) at the timepoints noted: vertical bars, 2 mV; horizontal bars, 2 ms.

Application of 40 µm MPEP (*n* = 9) for 20 min prior to HFT resulted in a significant impairment of both induction and expression of LTP (Fig. 2A; anova: within factor *F*_1,19_ = 10.367, *P* = 0.0001; between factor *F*_1,19_ = 1.806, *P* = 0.0373). Taking into account that activation of mGluR5 can modulate NMDAR-mediated currents (Mannaioni *et al*., 2001), we examined whether the same concentration of MPEP would affect LTP when applied after HFT. Here, we found that MPEP in a concentration of 40 µm (*n* = 8) also caused a significant impairment of LTP (Fig. 2A) in comparison to controls (anova: within factor *F*_1,23_ = 38.994, *P* = 0.0001; between factor *F*_1,23_ = 4.536, *P* = 0.0001). This effect did not derive from effects on basal synaptic transmission, which remained stable over the 4-h monitoring period and did not differ after MPEP treatment (40 µm, *n* = 9) when compared with control slices (*n* = 12; Fig. 2B). These data suggest that mGluR5 contributes to processes that underlie both the induction and late phases of LTP.

### Application of an mGluR1 antagonist prior to, but not after, LFS prevented LTD in the CA1 region

**Fig. 3:**
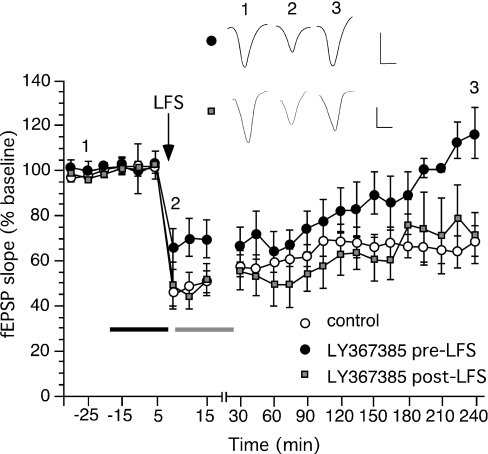
Application of an mGluR1 antagonist prior to, but not after, LFS prevented LTD in the CA1 region. LFS (2 Hz, 1200 pulses) induced persistent LTD which lasted for at least 4 h in the CA1 region *in vitro*. Application of the mGluR1 antagonist LY367285 (100 µm), for 20 min prior to LFS, significantly impaired the induction and expression phases of LTD. Application of the mGluR1 antagonist LY367285 (100 µm) for 20 min after LFS had no effect on the profile of LTD. Bar indicates drug application before (black) or after (grey) LFS. Insets: evoked potentials obtained in the presence of LY387385 applied pre-HFT or LY387385 applied post-HFT at the timepoints noted: vertical bars, 2 mV; horizontal bars, 2 ms.

Application of LY367385 (100 µm, *n* = 6) prior to LFS (2 Hz, 1200 pulses) resulted in a significant impairment of both LTD induction and expression (Fig. 3) compared to controls (*n* = 12; anova: within factor *F*_1,23_ = 28.7899, *P* = 0.0001; between factor *F*_1,23_ = 2.729, *P* = 0.0002). When LY367385 was applied after LFS (*n* = 7) no significant effect on LTD was evident (Fig. 3; anova: between factor *F*_1,23_ = 1.62, *P* = 0. 0.0514). These data suggest that antagonism of mGluR1 interferes with both the induction and late phases of LTD, and that mGluR1 must be active during the induction of LTD in order for persistent plasticity to occur.

### Application of an mGluR5 antagonist prior to LFS reduced the induction phase of LTD in the CA1 region, but LTD still occurred

**Fig. 4:**
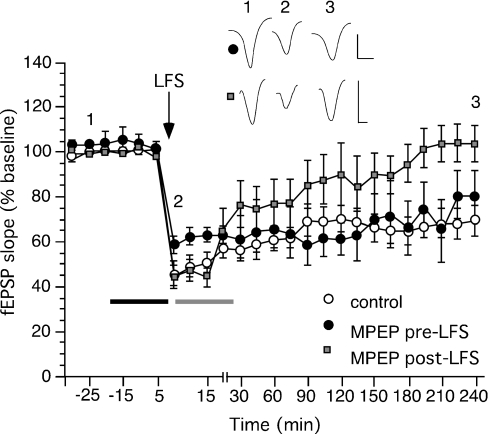
Application of an mGluR5 antagonist after LFS prevented LTD in the CA1 region. Prior antagonism reduced the LTD induction phase. LFS (LFS, 2 Hz, 1200 pulses) induced persistent LTD which lasted for at least 4 h in the CA1 region *in vitro*. Application of the mGluR5 antagonist MPEP (40 µm), for 20 min prior to LFS (LFS), significantly impaired the induction of LTD. However, a significant LTD still occurred compared to controls. Application of the MPEP (40 µm) for 20 min after LFS significantly inhibited the expression of LTD beyond 2 h post-LFS. Bar indicates drug application before (black) or after (grey) LFS. Insets: evoked potentials obtained in the presence of MPEP applied pre-HFT or MPEP applied post-HFT at the timepoints noted: vertical bars, 2 mV; horizontal bars, 2 ms and 1 ms for MPEP pre-LFS and MPEP post-LFS, respectively.

Application of 40 µm MPEP (*n* = 8) prior to LFS (2 Hz, 1200 pulses) resulted in a significant impairment of the LTD induction phase (Fig. 4; *P* < 0.05). However, persistent LTD still occurred and it was not significantly different from controls (*n* = 15; anova: within factor *F*_1,17_ = 0.03, *P* = 0.864; between factor *F*_1,17_ = 1.09, *P* = 0.363). This suggests that although antagonism of mGluR5 may result in a reduction in NMDA receptor currents (Mannaioni *et al*., 2001) or may change other excitability parameters such as the duration of the afterhyperpolarization (Ireland & Abraham, 2002), resulting in decreased depression, this is not sufficient to prevent stable LTD from occurring.

### Application of an mGluR5 antagonist after LFS prevented LTD

When MPEP (40 µm) was applied immediately after LFS (*n* = 13) a significant impairment of LTD occurred (Fig. 4) in comparison to controls (anova: within factor *F*_1,17_ = 2.39, *P* = 0.001; between factor *F*_1,17_ = 6.06, *P* = 0.0001). These data suggest that antagonism of mGluR5 interferes with the late phases of LTD.

### Agonist activation of mGluR5 converted STD into LTD

To verify that mGluR5 is important for late phases of synaptic plasticity we examined the effects of application of an mGluR5 agonist, CHPG, on short-term plasticity.

**Fig. 5:**
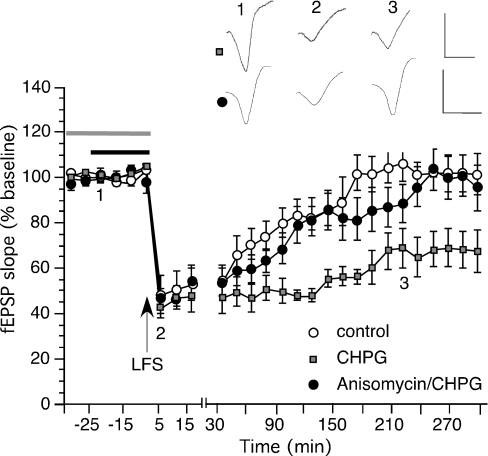
Application of an mGluR5 agonist facilitated STD into LTD in the CA1 region. Effects were prevented by a protein synthesis inhibitor. LFS (1 Hz, 900 pulses) induced STD which lasted for ∼ 90 min in the CA1 region *in vitro*. Application of the mGluR5 agonist CHPG (100 µm), for 20 min prior to LFS, significantly facilitated STD into LTD. Application of anisomycin (20 µm) for 30 min prior to LFS significantly prevented the facilitatory effects of CHPG on STD. Bar indicates drug application of CHPG (black) or anisomycin (grey) LFS. Insets: evoked potentials obtained in the presence of CHPG or CHPG and anismomycin at the timepoints noted: vertical bars, 2 mV; horizontal bars, 2 ms.

STD (*n* = 7) was evoked by LFS at 1 Hz (900 pulses). Treatment with CHPG (100 µm) prior to application of weak LFS resulted in persistent LTD (*n* = 8; Fig. 5). This finding supports an intrinsic role for mGluR5 in the persistency of hippocampal synaptic plasticity (anova: within factor *F*_1,21_ = 31.526, *P* = 0.0001; between factor *F*_1,21_ = 3.771, *P* = 0.001).

### Application of a protein synthesis inhibitor prevented facilitation of STD into LTD by mGluR5 agonist activation

Chemical LTD, induced by activation of group I mGluRs, is protein synthesis-dependent in the CA1 region (Huber *et al*., 2001). One possibility is that activation of protein synthesis comprises a mechanism underlying the regulation of late phases of synaptic plasticity. We therefore treated our slice preparation with the protein translation inhibitor anisomycin (20 µm, *n* = 8) prior to application of CHPG (100 µm) and weak LFS. Under these circumstances CHPG treatment did not convert STD into LTD (Fig. 5);, rather, evoked potentials returned to basal levels by ∼ 210 min after weak LFS (anova: CHPG + anisomycin vs. CHPG: within factor *F*_1,20_ = 9.884, *P* = 0.0001; between factor *F*_1,20_ = 3.951, *P* = 0.0001). This suggests that mGluR5 may trigger protein synthesis to enable persistency of synaptic plasticity.

## Discussion

In the present study we have shown that antagonism of mGluR1 and mGluR5 receptors impaired both the induction and late phases of both LTP and LTD in the CA1 region *in vitro*. MGluR1 effects occurred when receptor antagonism was implemented before plasticity was induced. MGluR5 antagonism, on the other hand, impaired LTP when applied either before or after HFT. Furthermore, mGluR5 antagonism did not prevent stable LTD when given prior to LFS, but prevented late phases of LTD when given after LFS. These data support, on the one hand, an important role for mGluR1 and -5 in the regulation of persistent synaptic plasticity in the CA1 region and, on the other hand, the idea that different mechanisms may facilitate the mediation of LTP and LTD by these receptors.

The involvement of group I mGluRs in synaptic plasticity in the CA1 region is not without controversy. Multiple papers have argued against a role for these receptors in CA1 plasticity, where studies *in vitro* have failed to identify a debilitation of either LTP or LTD following antagonist application (Chinestra *et al*., 1993; Manzoni *et al*., 1994; Selig *et al*., 1995; Thomas & O'Dell, 1995; Fitzjohn *et al*., 1999). Many of these studies were, however, conducted with MCPG which is an mGluR antagonist that may be most effective when the slice is naïve (Bortolotto *et al*., 1999). In contrast, more recent *in vitro* studies that have used subtype-specific group I mGluR ligands suggest a role for group I mGluRs in different phases of LTP (Raymond *et al*., 2000; Francesconi *et al*., 2004; Harney *et al*., 2006).

We were interested in addressing the issue of the involvement of group I mGluRs in synaptic plasticity that endures for several hours *in vitro* as, *in vivo*, a role for both mGluR1 and -5 in late phases of plasticity and in spatial learning have been described, both following pharmacological manipulations and in transgenic animals that lack either mGluR1 or mGluR5 (Aiba *et al*., 1994; Conquet *et al.*, 1994; Lu *et al*., 1997; Manahan-Vaughan, 1997; Balschun *et al*., 1999; Balschun & Wetzel, 2002; Naie & Manahan-Vaughan, 2004, 2005; Manahan-Vaughan & Braunewell, 2005). One possibility is that the dichotomy between *in vivo* and *in vitro* data derives from the duration of the observations: *in vivo* monitoring of synaptic plasticity typically is conducted for between 8 and 24 h after induction of plasticity. *In vitro* studies typically monitor synaptic plasticity for ∼ 60 min after induction. More to the point, impairments of synaptic plasticity following application of mGluR antagonists typically emerge ∼ 2 h after plasticity induction (Manahan-Vaughan, 1997; Naie & Manahan-Vaughan, 2004, 2005; Manahan-Vaughan & Braunewell, 2005). We therefore compared effects of antagonism of mGluR1 or mGluR5 over prolonged monitoring periods in the hippocampal slice preparation (up to 4 h) using drug concentrations that had been previously shown to elicit *in vitro* effects on CA1 excitability (Mannaioni *et al*., 2001). We found that LTP and LTD were impaired with effects typically becoming apparent ∼ 2 h after induction of synaptic plasticity. This is an important finding as it suggests that failure to observe lasting effects of mGluR antagonism in past *in vitro* studies may derive from the fact that the monitoring period was too short. This likelihood is corroborated by another study that examined the role of mGluR5 in CA1 synaptic plasticity: here, an effect on late phases of LTP was also identified (Francesconi *et al*., 2004).

As mentioned above, the potency of effects seen in our study may also derive from the subtype specificity of the antagonists used. Another significant point to mention is that the effectiveness of mGluR antagonists in preventing hippocampal synaptic plasticity may additionally relate to the relative recruitment of mGluRs by the plasticity-inducing protocol. Previous reports indicate that the participation of group I mGluRs in hippocampal LTP *in vitro* is influenced by the strength of the LTP-inducing tetanus (Wilsch *et al*., 1998). LTP induction that is strongly suprathreshold for activation of both NMDA receptors and voltage-gated calcium channels (VGCCs) does not depend upon activation of group I mGluRs (Wilsch *et al*., 1998). On the other hand, LTP that is induced by mildly suprathreshold stimulation which activates NMDA receptors but not VGCCs, and therefore depends more crucially on calcium release from intracellular stores, critically depends on mGluR activation (Wilsch *et al*., 1998).

Group I mGluRs couple positively to phospholipase C (PLC) via Gq proteins and are typically expected to mediate signalling processes through stimulation of diacylglycerol and inositol trisphosphate which trigger, respectively, stimulation of protein kinase C and calcium release from intracellular stores. However, they also mediate an increase in neuronal excitability that occurs independently of activation of PLC and inositol trisphosphate (Ireland & Abraham, 2002; Rae & Irving, 2004).

In the CA1 region mGluR1 also alters excitability via mechanisms that are distinct from those used by mGluR5. For example, mGluR1 mediates an increased frequency of spontaneous inhibitory postsynaptic potentials and a direct neuronal depolarization (Mannaioni *et al*., 2001). In the present study we observed that mGluR1 antagonism affects persistency of both LTP and LTD only if mGluR1 antagonism is implemented before induction of synaptic plasticity. This is in contrast to findings using a general group I mGluR antagonist (4CPG), where post-LFS application prevents persistent LTD (Manahan-Vaughan, 1997), and suggests that the effects seen with 4CPG may be mediated by antagonism of mGluR5, a possibility that is confirmed by our current data. A direct inhibition of NMDA receptor currents during plasticity induction as a consequence of mGluR1 antagonism may be one mechanism by which the impairment of LTP and LTD is seen (Skeberdis *et al*., 2001; Harney *et al*., 2006). A significant reduction in the LTD induction phase was evident in our study, suggesting that, at least for LTD, this may be an important mechanism.

MGluR1 activation mediates NMDA receptor cycling (Lan *et al*., 2001; Roche *et al*., 2001; Li *et al*., 2002). The impairments in plasticity that occurred when the antagonist was applied before the tetanus (or before LFS) may have been mediated by a run-down of NMDA receptors or increased receptor internalization, which in turn would alter the longevity of synaptic plasticity. Effects on LTD may also be mediated by regulation of fast transient and persistent Na^+^ currents (Carlier *et al*., 2006), and of calcium signalling in interneurons (Topolnik *et al*., 2006). Not only was late plasticity inhibited when mGluR1 was antagonized during the plasticity induction protocol, but marked reductions in the amplitude of the induction (early) phase of plasticity were seen. This is in line with previous observations of the effects of mGluR1 antagonism in the hippocampus *in vivo* (Naie & Manahan-Vaughan, 2005; Naie *et al*., 2007). The loss of late LTP in the presence of the mGluR1 antagonist does not derive simply from the weaker LTP induction, however; under control conditions, the induction of LTP with a similar (small) amplitude does not preclude persistent LTP that lasts for > 4 h in freely moving rats (Naie & Manahan-Vaughan, 2005).

MGluR5 antagonism affected LTP regardless of whether the antagonist was given before or after the tetanus. When MPEP was applied before the tetanus a marked impairment of LTP induction was evident. Activation of mGlu5 results in suppression of the calcium-activated potassium current (*I*_AHP_) and potentiation of NMDA receptor currents (Jia *et al*., 1998; Attucci *et al*., 2001; Mannaioni *et al*., 2001) In the hippocampal slice preparation, antagonism of mGluR5 receptors results in an impairment of LTD induction due to an inhibition of mGluR5-mediated NMDAR currents (Harney *et al*., 2006) and subsequent alteration of intracellular calcium levels (Harney *et al*., 2006; Naie *et al*., 2007) This mechanism is a probable explanation for the effects we also saw on induction processes.

The inhibition of late phases of LTP that occurred when MPEP was given after the tetanus suggests that mGluR5 facilitates persistent LTP by additional mechanisms. A time window has been reported for the post-tetanic facilitation, by the group I agonist DHPG, of STP into LTP (Manahan-Vaughan & Reymann, 1996). Metabotropic glutamate receptors alter their expression as a consequence of induction of synaptic plasticity (Manahan-Vaughan *et al*., 2003). Furthermore, activation of mGluR5 results in the stimulation of dendritic protein synthesis to enable LTD (Huber *et al*., 2001), and group I mGluRs trigger *de novo* protein synthesis to enable long-lasting LTP (Raymond *et al*., 2000). We observed in the present study that application of a protein synthesis inhibitor prevented the facilitation of STD into LTD that is enabled by application of an mGluR5 agonist. Thus it may be that the impairment of later phases of synaptic plasticity, that we observed in the presence of MPEP, derive from a disruption of processes that underlie protein synthesis.

Curiously, application of MPEP prior to LFS impaired the induction of LTD but did not prevent persistent LTD from being expressed. In contrast, application of the antagonist after LFS prevented late LTD. These effects may derive from the temporal dynamics of mGluR5 facilitation of LTD. It is possible that mGluR5 must be active immediately after LFS in order for persistent LTD to occur. This could relate to a lingering spillover of glutamate after conclusion of LFS that activates perisynaptically localized mGluR5 (Lujan *et al*., 1996), or to the involvement of constitutive activity of mGluR5 in the persistence of LTD (Joly *et al*., 1995).

The bidirectional modulation of synaptic plasticity by mGluR1 has interesting implications for information storage in the CA1 region. LTP and LTD appear to engage in the encoding of different functional aspects of spatial memory (Manahan-Vaughan & Braunewell, 1999; Kemp & Manahan-Vaughan, 2004, 2005, 2007; Uzakov *et al*., 2005; Etkin *et al*., 2006). Aside from differences in the regulation of LTP and LTD in the CA1 region, subregional differences in the regulation of synaptic plasticity by group I mGluRs also occur (Naie *et al*., 2007). Regulation by group I mGluRs of LTP and LTD is determined by the frequency of afferent activity as well as the intracellular calcium signal generated by activation of the receptor (Harney *et al*., 2006; Naie *et al*., 2007). The particular pattern of mGluR activation may be a key mechanism in the determination of which types of synaptic plasticity are generated in response to incoming sensory information, and which type of memory ultimately results.

The results of this study demonstrate that activation of both mGluR1 and mGluR5 is critically required for persistent LTP and LTD in the hippocampal CA1 region. Whereas activation of mGluR1 during the plasticity-inducing protocol is essential for persistent bidirectional plasticity, the time window for activation of mGluR5 to enable LTP or LTD extends to periods after cessation of the plasticity-inducing protocol. Furthermore, the regulation by mGluR5 of late phases of plasticity appears to be mediated by stimulation of protein synthesis. These data emphasize an important functional role for group I mGluRs in the regulation of hippocampal synaptic plasticity and highlight the importance of this receptor for information storage in the CA1 region.

## Abbreviations

CHPG: (R,S)-2-chloro-5-hydroxyphenylglycine
DHPG: dihydroxyphenylglycine
LFS: low-frequency stimulation
HFT: high-frequency tetanization
LTD: long-term depression
LTP: long-term potentiation
LY367385: (S)-(+)-α-amino-4-carboxy-2-methylbenzene-acetic acid
mGluR: metabotropic glutamate receptor
MPEP: 2-methyl-6-(phenylethynyl)pyridine
NMDA: *N*-methyl-d-aspartate
PLC: phospholipase C
STD: short-term depression
STP: short-term potentiation.
